# Top down and bottom up selection drives variations in frequency and form of a visual signal

**DOI:** 10.1038/srep09543

**Published:** 2015-04-17

**Authors:** Chien-Wei Yeh, Sean J. Blamires, Chen-Pan Liao, I.-Min Tso

**Affiliations:** 1 Department of Life Science, National Chung-Hsin University, Taichung 40227, Taiwan; 2 Department of Life Science, Tunghai University, Taichung 40704, Taiwan; 3 Evolution & Ecology Research Centre, School of Biological, Earth & Environmental Sciences, The University of New South Wales, Sydney 2052, Australia

## Abstract

The frequency and form of visual signals can be shaped by selection from predators, prey or both. When a signal simultaneously attracts predators and prey, selection may favour a strategy that minimizes risks while attracting prey. Accordingly, varying the frequency and form of the silken decorations added to their web may be a way that *Argiope* spiders minimize predation while attracting prey. Nonetheless, the role of extraneous factors renders the influences of top down and bottom up selection on decoration frequency and form variation difficult to discern. Here we used dummy spiders and decorations to simulate four possible strategies that the spider *Argiope aemula* may choose and measured the prey and predator attraction consequences for each in the field. The strategy of decorating at a high frequency with a variable form attracted the most prey, while that of decorating at a high frequency with a fixed form attracted the most predators. These results suggest that mitigating the cost of attracting predators while maintaining prey attraction drives the use of variation in decoration form by many *Argiope* spp. when decorating frequently. Our study highlights the importance of considering top-down and bottom up selection pressure when devising evolutionary ecology experiments.

Animals use visual signals in a multitude of ways^1^. For example, prey can deter predators using aposematic colouration^2^. Predators, on the other hand, may use visual signals to exploit a preexisting visual bias of their prey to lure them towards themselves or into a trap^3^,^4^. While predators benefit by using deceptive signals, prey may learn to avoid them and apply counter-selection^5^. Such prey and predator counter adaptations have been described as evolutionary arms races^6^,^7^. The arms race analogy, nonetheless, is a prohibitively simple description of the co-evolutionary dynamics between predators and prey. In reality there are multiple interacting predators and prey as well as secondary predators and parasites and the foraging strategies of predators are usually driven by a combination of top down (from predators or parasites) and bottom up (from prey) selection pressure^8^,^9^. Researchers may use game theory to make sense of the strategies employed by predators facing multiple selection pressures^10^,^11^.

Approximately 22 genera of diurnal orb web spiders add some form of silken decoration (also called stabilimenta) to their webs. The costs and benefits of the decorations are widely debated (see reviews by Herberstein et al., Bruce, and Walter and Elgar^12^,^13^,^14^). One hypothesis suggests that decorations are used as a deceptive signal that lures insects toward the web by mimicking cues the insects use to search for food^12^,^13^,^15^,^16^. This same hypothesis postulates that when decorations are used as prey attractants they may also lure unwanted bird and wasp predators and parasites^12^,^16^,^17^,^18^,^19^,^20^.

Many spiders of the genus *Argiope* construct decorations that consist of four silk bands arranged in a cruciate (x-shaped) form^16^,^21^. An individual spider might build a web with all four or 3, 2, 1, or no decoration bands added on any given day (Figure 1)^5^,^22^,^23^,^24^,^25^. Accordingly, the form and frequency of web decorations will vary considerably between individuals over space and within individuals over time. The consequence of such variability over space and time is that the prey and predators of the spiders should repeatedly encounter different decoration forms each time they visit a spider web^26^. Subsequently, it might be hypothesized that decoration variability has foraging and predation consequences for the spiders.

Bees and wasps can identify sites that provide food rewards by identifying, remembering and visually discriminating among objects of different shapes and colours^27^,^28^,^29^. It is thus plausible that bees and wasps can, in certain contexts^30^, visually identify and learn to avoid potentially lethal objects such as spiders and spider webs^5^,^29^. *Argiope* spiders may, accordingly, vary the form and frequency of their web decorations to prevent bees or wasps associating their web decorations with a spider's presence^5^,^20^,^31^,^32^. Factors other than prey and predators, e.g. nutrient availability, light levels, disturbance and molting^22^,^25^,^31^,^33^, nevertheless, independently induce variability in web decoration forms. Differentiating between the drivers and constraints of web decoration variability has thus proven notoriously difficult^12^,^13^,^14^.

Here we aimed to determine the top down and bottom up drivers of web decoration variability in *Argiope* spiders using dummy spiders and decorations that were colour matched, in the eyes of bees and wasps, and real *Argiope* *aemula* spiders and their decorations (see Ref. 16 for detailed descriptions). This enabled us to discern the predator and/or prey attraction consequences of temporal variations in decoration frequency and form by examining the foraging and predation consequences of a simulated game where spiders choose among decorating webs at high or low frequencies combined with fixed or variable forms.

## Results

### Influence of decorating strategy on prey attraction

**Figure 2:**
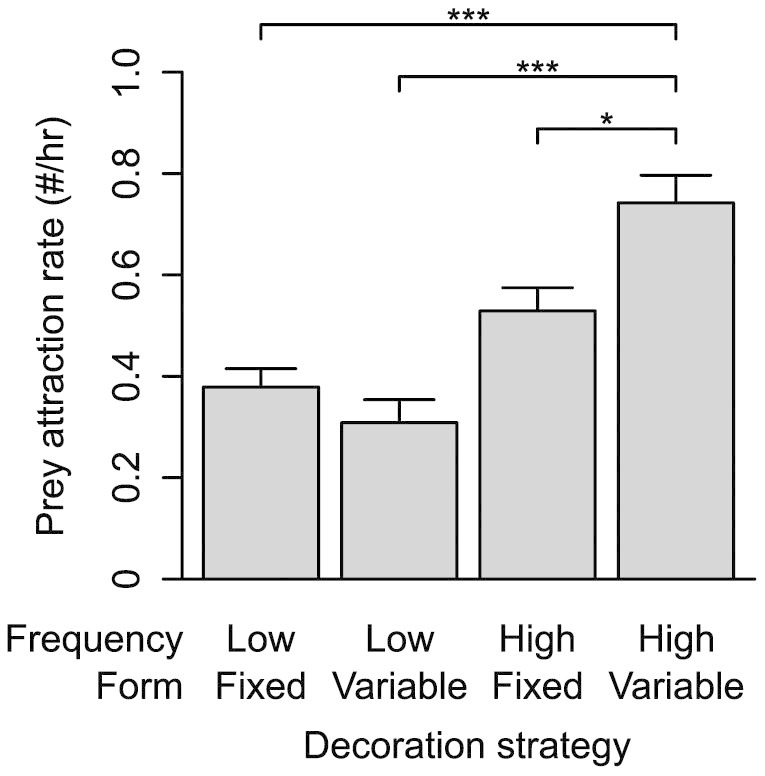
Mean ( SEM) prey attraction rates. Shows mean values ( SEM) and results of a Negative Binomial Regression analysis (* indicates significant differences at *P* <0.05, and *** indicates significant differences at *P* < 0.01), for prey attraction rates for the four decorating strategies.

We first determined prey attraction rates for four decorating strategies: (1) decorating webs at a low frequency (twice over the eight days) with a fixed form, (2) decorating webs at a low frequency with a varying form, (3) decorating webs at a high frequency (eight times over eight days) with a fixed form, and (4) decorating webs at a high frequency with a varying form, and found a significant difference among them (Negative binominal regression, ^2^ = 90.14, *P* < 0.0001). Hymenopterans (~30%), dipterans (~25%) and lepidopterans (~35%) were the predominant insects attracted in all instances. We conducted pair-wise comparisons between low and high frequency strategies and found that the strategy of decorating at a high frequency with varying form attracted significantly more prey than all other strategies (Table 1). Of the other strategies, the low frequency with fixed form and low frequency with variable form decorating strategies attracted fewer prey than the high frequency with fixed form strategy (Figure 2). Our findings thus suggest that *Argiope* web decorations are attractive to prey but the consistent use of fully cruciate decorations does not maximize the prey attraction rate.

### Influence of decorating strategy on predator attraction

**Figure 3:**
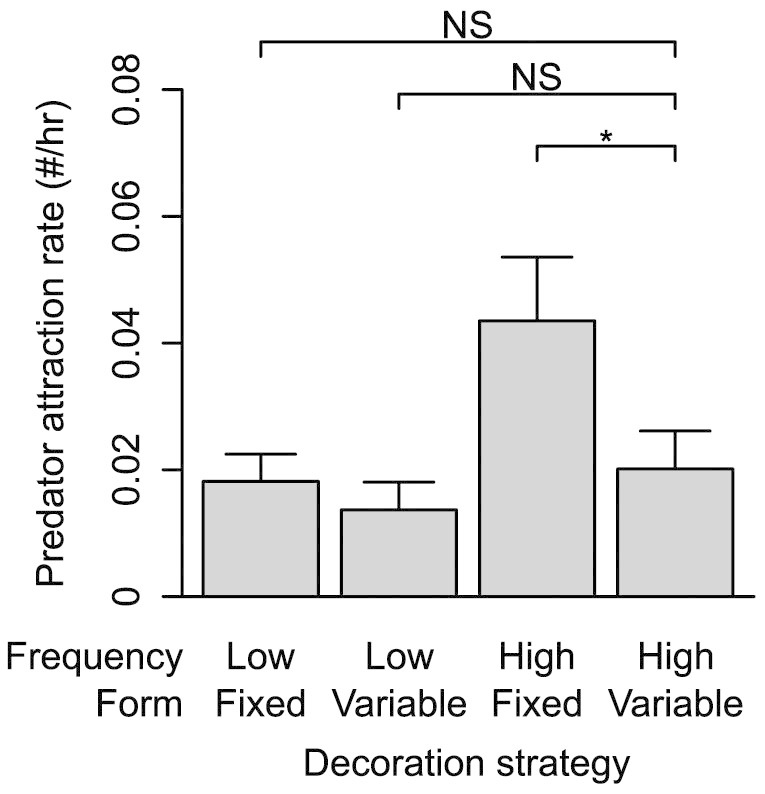
Mean ( SEM) predator attraction rates. Shows mean values ( SEM) and results of a Generalized Linear Poisson Regression analysis (* indicates significant differences at *P* <0.05), for predator attraction rates for the four decorating strategies.

We, secondly, tested whether the four strategies influenced the rate of predator attraction to artificial *Argiope* *aemula* webs. All observed predators were wasps. The high frequency and fixed form decorating strategy attracted significantly more predators than any of the other three strategies, all of which had similar predator attraction rates (Figure 3; Table 2). These finding agree with other studies^18^,^19^,^20^ that show *Argiope* web decorations attract predators in addition to prey. Decorating at a high frequency with a variable form attracted fewer predators than decorating at a high frequency with a fixed form. Mitigating the costs of attracting predators while attracting prey, therefore, seems to be a driver of decoration form variation in *A. aemula*.

## Discussion

Spiders of the genus *Argiope* are the most ardent decorators; with most of its 70+ species decorating their webs with some form of conspicuous silk bands^12^,^14^,^16^. The reason for so much inter- and intra-specific variation in *Argiope* decoration frequency and form, however, has puzzled researchers for over a century. We showed that varying the form and frequency of decorations promotes a fitness payoff to individuals, thus providing an ultimate explanation for decoration frequency and form variations. Factors other than prey and predators, e.g. nutrient availability, light levels, web damage and molting^20^,^22^,^23^,^24^,^25^,^34^, may act as additional cues or constraints that enhance or reduce the frequency or form variations in some species. The confounding effect of these cues or constraints may explain why many *Argiope* spp. in the wild decorate their webs at different frequencies or use a wider range of forms than we depict herein.

We experimentally assessed the top down (predator attraction) and bottom up (prey attraction) consequences of different web decorating strategies. Decorating the web at high frequency but with variable forms is the strategy with the greatest overall payoff for *A. aemula*. Laboratory studies have shown that the mitigation of bee and wasp recognition of web decorations is a reason why *Argiope* spiders build webs with a high degree of variability^5^,^16^,^26^. Another study^35^ simultaneously exposed trained stingless bees to real *Argiope keyserlingi* webs containing four different decorations forms and found that all of the webs intercepted the same number of bees. We, nevertheless, used artificial webs (thus controlling for background noise induced by variations in web size and geometry), and varied decoration forms over time, and found that the strategy of high frequency decorating with a variable form attracted more prey than that of high frequency decorating with a fixed form. Our finding accordingly suggests that bees and wasps may avoid webs in the field where similar decoration forms are repeatedly used by spiders.

We found that the majority of bees that were attracted to the decorations flew toward them using a stereotypical side-to-side beescanning behaviour^36^,^37^ before flying away. This scanning behaviour may enable bees to readily recognize and subsequently avoid frequently used decoration forms^29^,^37^,^38^. We expect other prey insects to have some ability to recognize and avoid frequently used decoration forms. We found that predatory wasps were mostly attracted to the artificial webs that were decorated at a high frequency with a fixed form. Wasps also have an innate ability to forage by the selective association of stimuli^17^,^39^,^40^. This ability may be a reason why we found predatory wasps to be attracted to frequently decorated artificial webs with a fixed form. The high decorating frequency and fixed form strategy thus seems to come at the dual costs of bees learning to avoid the webs due to their frequent exposure to a particular decoration form and the attraction of predatory wasps. Accordingly, it appears to be the least rewarding strategy.

Many features of spider webs, including their two- or three-dimensionality, the use of specific silks for specific purposes, and the use of decorations and other components, have been hypothesized to have evolved as a consequence of co-evolutionary arms races between spiders and their insect prey and/or predators^12^,^13^,^41^,^42^,^43^. The life-dinner principle of predator-prey arms races states that the evolutionary pressure on prey to avoid predation is stronger than the evolutionary pressure on predators to attain a meal. Accordingly, traits under simultaneous top down and bottom up selection pressure should be more susceptible to top-down (i.e. predatory) than to bottom-up selection pressure^6^,^7^. Under this principle it seems inexplicable why *Argiope* should decorate their webs at all. Indeed, many orb web spiders can survive without the use of decorations as lures. It has been hypothesized that web decorations are a pleisiomorphic trait among web-building spiders which has become redundant in many clades, perceptibly under top down selection pressure^12^,^16^,^44^. In *Argiope* spp. that add cruciate decorations to their webs, however, an alternative strategy may have evolved to minimize predation pressure; the retention of decorating but with high variation in decorating frequency and form.

In summary, we simulated a game of spiders choosing among decorating webs at high or low frequencies using fixed or variable forms to determine the top down and bottom up drivers of spider web decorating variation. Our results suggest that that the need to avoid predation while attracting prey primarily drives variations in spider web decorating frequency and form. We speculate that varying the decoration form and frequency simultaneously prevents prey from associating them with danger while preventing predators from generating a search image based on their experience encountering particular decoration forms. More studies are required to ascertain whether top-down and bottom-up selective pressures influence the expression of various other visual signals, such as sexual signals.

## Methods

### Species used and study site

Adult female St Andrew's cross spiders, *Argiope aemula* Walckenaer 1841 (Araneae: Araneidae) are a large-bodied (adult body length ~25mm) orb web spider with conspicuous body colouration that constructs vertical orb webs near the ground in grass and shrubs and builds a cruciform silk decoration on its web. Our study was conducted from July through August 2010 at a forest edge (the typical habitat of *A. aemula*) at Lien-Hwa-Chih Research Center (1205236 E, 235513 N), Yu-Chi, Nantou County, Taiwan. The site is dominated by *Bidens pilosa* var. *radiate* and *Mimosa diplotricha* plants.

### Dummy spiders and decorations

Colour controlled dummy spiders were made by cutting black paper into the size and shape of a spider's body and legs. Yellow paint was used to add the yellow body stripes characteristic of this species (Supplementary figure S1) onto the dummies. Dummy decorations were constructed by cutting white paper into the shape of decorations measured in the wild (~30mm long 5mm wide). To control for background visual noise induced by variations in web size and geometry the dummies were attached using water proof glue onto an artificial web consisting of 12 strands of 600mm long green string (colour matched to the background vegetation using the protocol of Cheng et al.^16^) running horizontally between two 900mm long wooden stakes placed 500mm apart. The string was attached to the top portion of the stakes by masking tape ensuring all dummies were placed approximately 600mm off the ground (Supplementary figure S2). We replaced any tape that failed to keep the strings taught. All dummies were constructed within one day of being used in the experiment and they were only used once.

To ensure the dummy spiders and decorations resembled real spider bodies and decorations when viewed by wasp predators and bee prey, we measured the spectral reflectance functions of the: (i) yellow paint, (ii) silver, black and white paper, (iii) the dorsal abdominal bands, and (iv) the yellow and black stripes on the ventrum, of three randomly collected spiders (Supplementary figure S3) using a spectrometer and OOIBase32 analytical software (S4000, Ocean Optics, Inc., Dunedin, FL, USA) connected to a 450W Xenon arc lamp.

The spectrometer was standardized using a high reflectivity standard (STAN-SSH). We used published spectral reflectance functions for: (i) *A. aemula* decorations and bodies contrasted against a typical vegetative background (*Is*(**))^16^, (ii) spectral sensitivities of honeybee photoreceptors (*S*(**)), the only hymenopteran insects with photoreceptor sensitivities mapped across the 300700nm waveband^19^, and (iii) the daylight illumination (*D*(**)) at the study site^45^, to determined bee UV, blue and green photoreceptor excitation indices (*P*) using the formula: 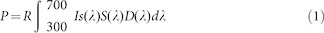
The sensitivity factor, *R*, was calculated using the equation:
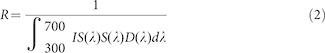
The photoreceptor excitation indices were plotted onto a colour hexagon derived for honeybee vision^46^ and chromatic contrast values were calculated as the Euclidean distances between the excitation indices in hexagon units. The achromatic contrasts were calculated as the excitation values of the honeybee green receptors when viewing *A. aemula*'s decorations and bodies contrasted against the vegetation background divided by those when viewing only the vegetation background. A photoreceptor discrimination threshold value of 0.1 hexagon units was assumed. There are reports of honeybee colour discrimination thresholds as low as 0.04 hexagon units for differentially conditioned bees, but we considered 0.1 to be appropriate to use because it accounts for the different sensitivities of the UV, blue and green photoreceptors of absolute conditioned or unconditioned bees^47^. Although we expected a diversity of insect taxa to be attracted to the artificial webs, only for hymenopteran insects are neuroethological visual models established. We thus had no choice but to use a honeybee model as a surrogate to quantify how the colour paper and silk decorations were viewed by insects in general. The honeybee model is directly applicable to wasps since they have similar types of photosensitive cells, occur in similar environments, and are phylogenetically related.

We measured the spectral reflectance functions of the black, silver and white paper and yellow paint and determined honeybee photoreceptor indices and chromatic and achromatic contrast values for each as described above. One-tailed *t* tests comparing real and dummy spiders and decorations with each other and with the photoreceptor discrimination threshold found both the yellow spider body parts and yellow paint and the web decorations and white paper to be chromatically indistinguishable to bees and above the photoreceptor discrimination threshold (Supplementary Table 1). The colour signals of dummy spiders thus resembled those of real spiders in the eyes of bees, wasps and, presumably, other insects. The chromatic contrast values of the white paper and the silk decorations were significantly lower than the discrimination threshold (Supplementary Table 1). Thus, the paper and decorations should appear similar when viewed by insects.

### Experiment

We performed an experiment in July and August of 2010 to test whether prey and predator attraction varied when spiders decorate their webs frequently or infrequently with consistent or varying forms. Four strategies were simulated: (1) decorating webs at a low frequency (twice over the eight days) with a fixed form, (2) decorating webs at a low frequency with a varying form, (3) decorating webs at a high frequency (eight times over the eight days) with a fixed form, and (4) decorating webs at a high frequency with a varying form, over eight consecutive days. Dummy spiders and decorations described in the previous section were used to simulate the above four strategies. We performed a pilot survey of the decorating frequencies of individual *A. aemula* at our study area and found individuals to decorate their webs between two and eight times over any eight day period using predominantly (>95% of the time) a cruciform and a two-armed, diagonally aligned (i.e. / shaped) form. The decorating frequencies used thus reflected the range of decorating frequencies observed at our study area^16^. In all instances the fixed form was fully cruciform, as these are the form most commonly built by *A. aemula*^15^,^16^ and the alternative form was a two-armed, diagonally aligned (/ shaped) form. For the varying form strategy we alternated daily between the use of the fully cruciform and the two-armed form. Simultaneous strategies (*N* = 20) were randomly distributed throughout the study area, placed approximately ~30m apart (to minimize the chance that individual bees will visit each set up), and faced random, unspecified, directions. Each experiment lasted eight days and was repeated three times.

We placed video cameras (Sony TRV 118 Hi-8 and Sony HDD) ~1m from the artificial webs and all were monitored between 0800 and 1100h, or between 1100 to 1400h, over eight days. We viewed the 2671hours of video footage in the laboratory at Tunghai University, Taichung, and recorded the number of times that prey (classified as flies, bees, moths or smaller unidentified insects) or predators (all were wasps) flew directly at and to within ~1cm of the spider or artificial web. The artificial webs were all set up in front of dense vegetation so all insects were observed approaching the webs from the same side as the cameras, facilitating clear images of all insect interactions. As unforeseen circumstances and minor technicalities resulted in there being differences in the hours of footage available among the four strategies (i.e. 661, 644, 657 and 709hours of footage were collected for the low frequency with a fixed form, low frequency with a varying form, high frequency with a fixed form, and high frequency with a varying form, decorating strategies respectively), we determined prey and predator attraction rates as the number of prey or predator interactions per hour of monitoring.

### Statistical analyses

All of the prey attraction data fitted a negative binomial model (Pearson ^2^ = 169.91, *P* = 0.1521), so a two-factor Negative Binomial Regression analysis was performed with prey attraction rate designated the dependent variable, strategy the independent variable, and hours of footage the offset variable. Due to excessive zeroes the predator attraction rates fitted a Poisson distribution (Pearson ^2^ = 170.64, *P* = 0.144), so a Generalized Linear Poisson Regression model was used to compare the predator attraction rates for the different strategies with predator attraction rate designated the dependent variable, strategy the independent variable and hours of footage the offset variable. Pearson goodness-of-fit tests were used to compare the Regression models with a null model. All analyses were performed using SAS, version 9.2 (SAS Foundation for Statistical Computing, Raleigh, North Carolina).

## Author Contributions

C.W.Y. conducted the field research. C.W.Y. and C.P.L. conducted the spectral analyses. S.J.B. and I.M.T. wrote the main text. All authors contributed to the data analysis and reviewed the manuscript.

## Supplementary Material

Supplementary Information Supplementary information

## Figures and Tables

**Figure 1:**
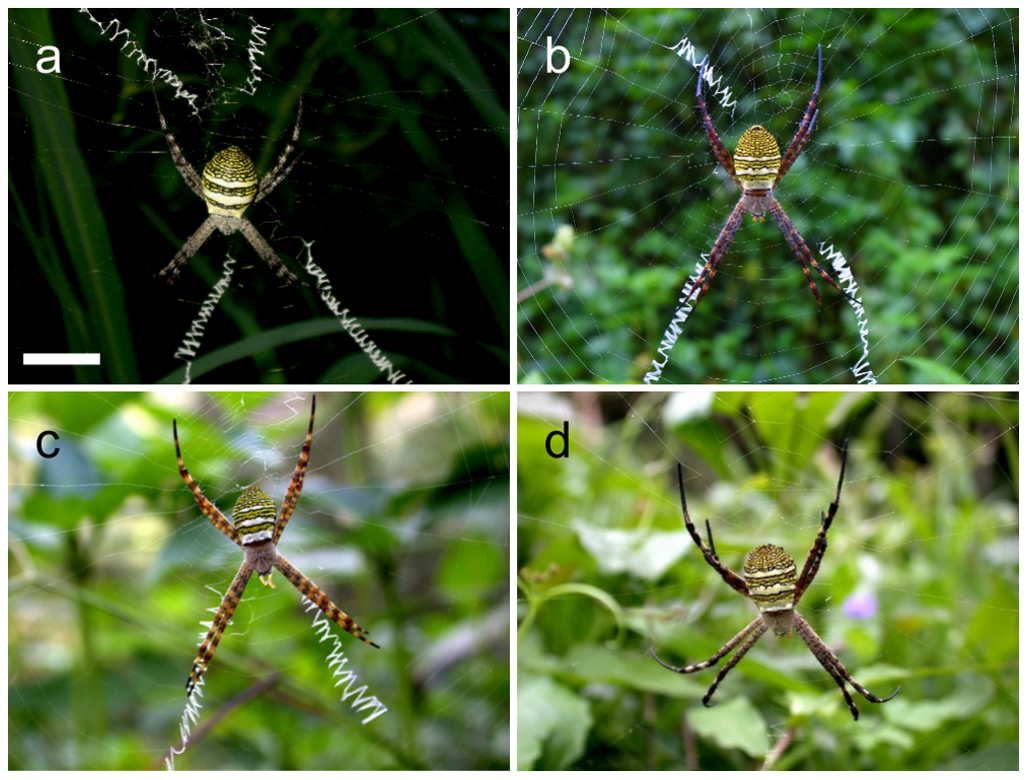
*Argiope aemula* web decoration variability. Shows female *Argiope aemula* on webs with (a) four, (b) three, (c) two, or (d) no decoration bands. (Scale bar = 20mm). Photographs in (a) and (b) were taken by C.-P. L. Photographs in (c) and (d) were taken by C.-W. Y.

**Table 1:** Prey attraction rate fitted with a negative binomial regression. A Pearson **^2^ goodness-of-fit test shows that this model reasonably fits the data (**^2^ = 115.5, df = 102, p = 0.1710). The ** of the high frequency variable form group was arbitrarily designated as 0 to facilitate comparison of probabilities of different events. The ratio between probabilities of two certain events was *e*

Coefficient	**	Standard error	*Z*	*P*
Intercept	0.7701	0.0487	15.823	< 0.0001
Low frequency fixed form	0.6699	0.1338	5.005	< 0.0001
Low frequency variable form	0.8793	0.1382	6.361	< 0.0001
High frequency fixed form	0.3356	0.1336	2.512	0.012
High frequency variable form	0	_	_	_

**Table 2:** Predator attraction rate fitted with a negative binomial regression. A Pearson **^2^ goodness-of-fit test shows that this model reasonably fits the data (**^2^ = 98.5, df = 102, p = 0.5797). The ** of the high frequency variable form group was arbitrarily designated as 0 to facilitate comparison of probabilities of different events. The ratio between probabilities of two certain events was *e*

Coefficient	**	Standard error	*Z*	*P*
Intercept	3.8392	0.1424	26.953	< 0.0001
Low frequency fixed form	0.0960	0.4065	0.236	0.8134
Low frequency variable form	0.3927	0.4465	0.879	0.3792
High frequency fixed form	0.7467	0.3548	2.105	0.0353
High frequency variable form	0	_	_	_
