# Supplementary material for: Top down and bottom up selection drives variations in frequency and form of a visual signal
Source: Sci Rep. 2015 Apr 17;5:9543. doi: 10.1038/srep09543 (PMC4406052; doi:10.1038/srep09543)
Supplement: Supplementary Information [file srep09543-s1.pdf]

Top down and bottom up selection drives variations in frequency and form of a visual signal

[Supplementary information]

Chien-Wei Yeh[1], Sean J Blamires[2,3], Chen-Pan Liao[2] and I-Min Tso[1,2]

[1]Department of Life Science, National Chung-Hsin University, Taichung 40227, Taiwan.
[2]Department of Life Science, Tunghai University, Taichung 40704, Taiwan.
[3]Evolution & Ecology Research Centre, School of Biological, Earth & Environmental Sciences, The University of New South Wales, Sydney 2052, Australia.

Correspondence and requests for materials should be sent to I.M.T. (spider@thu.edu.tw).

Supplementary Table S1. Mean (± SEM) chromatic contrast values of color paper and paint used for dummies compared to their corresponding spider body coloration and decorations by honey bees, and the results of one-sample *t* tests comparing the various values with a honeybee photoreceptor discrimination threshold of 0.1 hexagon units.

**Comparisons**	**Chromatic contrast values**	***t***	**df**	***p***
Silver paper vs. spider silver band	0.0501 ±0.0045	0.04	41	0.45
Black paper vs. spider black band	0.0529 ± 0.0040	0.71	59	0.24
Yellow paint vs. spider yellow band	0.2552 ± 0.0103	19.77	23	< 0.0001
White paper vs. web decoration	0.0452 ± 0.0017	-2.78	39	0.0042

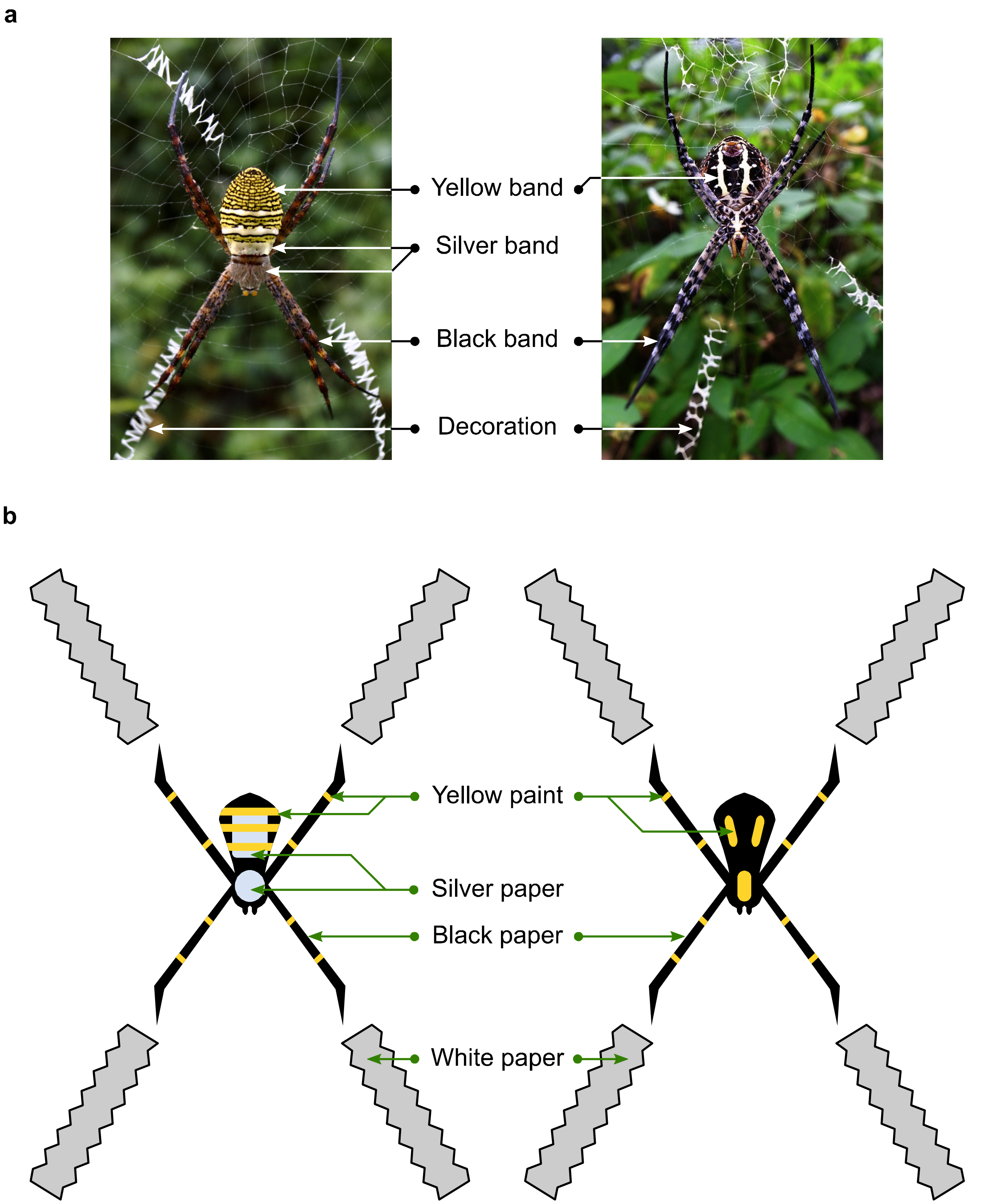

Supplementary Figure S1. (a) Dorsal (left) and ventral (right) view of female *Argiope aemula* against a web decoration. Photographs taken by C.-P. L. (b) A schematic representation of the dorsal and ventral view of the paper dummies resembling female *Argiope aemula* in size, appearance and colour used in our field experiments.

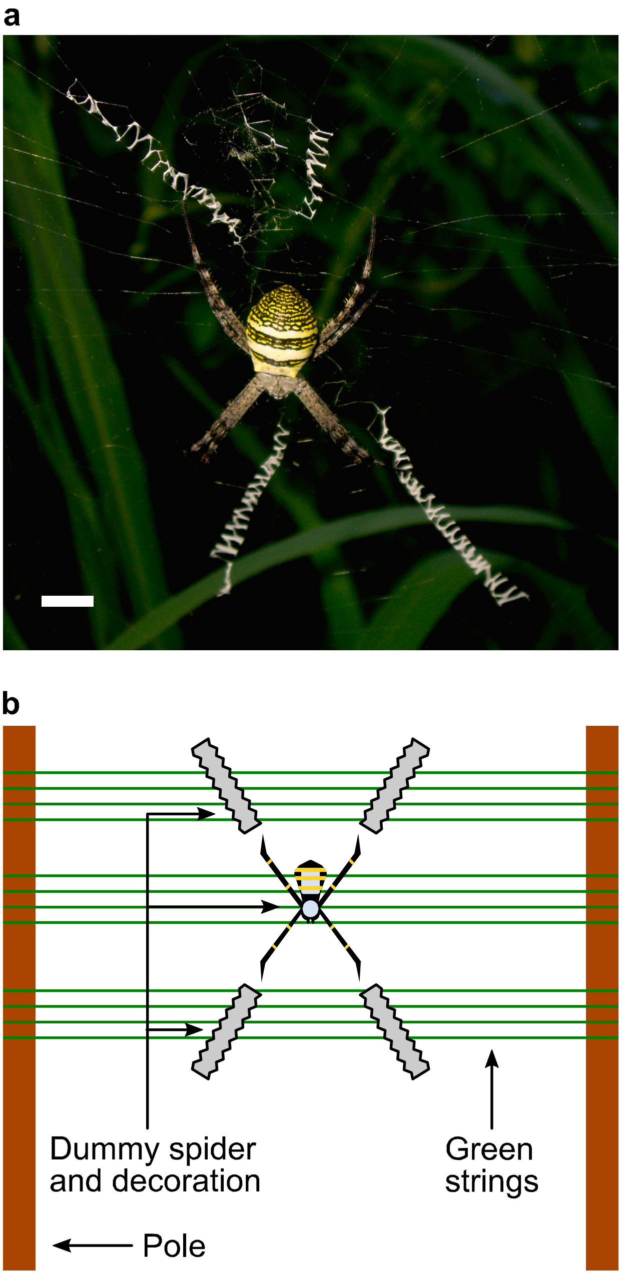

Supplementary Figure S2. Details of our experimental setup. Shows: (a) a live female *Argiope aemula* with a fully cruciform decoration. Photograph taken by C.-P. L. (b) A diagrammatic representation of the setup of a dummy spider against a fully cruciform dummy decoration.

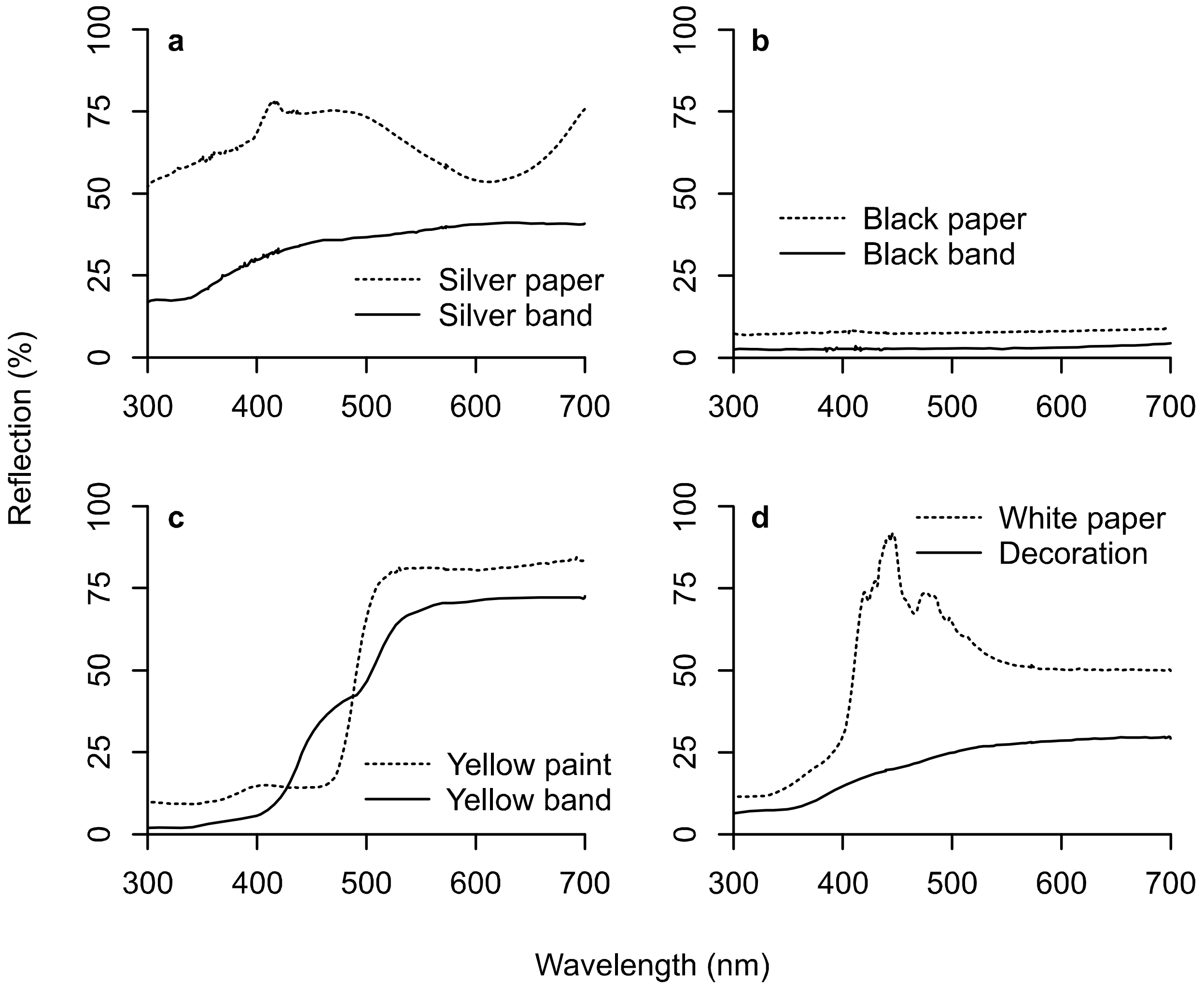

Supplementary Figure S3. Reflectance spectra of silver paper (a), black paper (b), yellow paint (c) and white paper (d) used to construct dummies and the corresponding spider body parts and web decorations.
